# FDG PET/CT parameters and correlations with tumor-absorbed doses in a phase 1 trial of ^177^Lu-lilotomab satetraxetan for treatment of relapsed non-Hodgkin lymphoma

**DOI:** 10.1007/s00259-020-05098-x

**Published:** 2020-11-16

**Authors:** Ayca Løndalen, Johan Blakkisrud, Mona-Elisabeth Revheim, Ulf Erik Madsbu, Jostein Dahle, Arne Kolstad, Caroline Stokke

**Affiliations:** 1 Division of Radiology and Nuclear Medicine, Oslo University Hospital, Oslo, Norway; 2 Faculty of Medicine, University of Oslo, Oslo, Norway; 3 Department of Physics, University of Oslo, Oslo, Norway; 4 Nordic Nanovector ASA, Oslo, Norway; 5 Department of Oncology, Radiumhospital, Oslo University Hospital, Oslo, Norway

**Keywords:** Non-Hodgkin lymphoma, Radioimmunotherapy, ^177^Lu-lilotomab satetraxetan, Tumor dosimetry, FDG PET/CT

## Abstract

**Purpose:**

^177^Lu-lilotomab satetraxetan targets the CD37 antigen and has been investigated in a first-in-human phase 1/2a study for relapsed non-Hodgkin lymphoma (NHL). Tumor dosimetry and response evaluation can be challenging after radioimmunotherapy (RIT). Changes in FDG PET/CT parameters after RIT and correlations with tumor-absorbed doses has not been examined previously in patients with lymphoma. Treatment-induced changes were measured at FDG PET/CT and ceCT to evaluate response at the lesion level after treatment, and correlations with tumor-absorbed doses were investigated.

**Methods:**

Forty-five tumors in 16 patients, with different pre-treatment and pre-dosing regimens, were included. Dosimetry was performed based on multiple SPECT/CT images. FDG PET/CT was performed at baseline and at 3 and 6 months. SUV_max_, MTV, TLG, and changes in these parameters were calculated for each tumor. Lesion response was evaluated at 3 and 6 months (PET_3months_ and PET_6months_) based on Deauville criteria. Anatomical changes based on ceCT at baseline and at 6 and 12 months were investigated by the sum of perpendiculars (SPD).

**Results:**

Tumor-absorbed doses ranged from 35 to 859 cGy. Intra- and interpatient variations were observed. Mean decreases in PET parameters from baseline to 3 months were ΔSUV_max-3months_ 61%, ΔMTV_3months_ 80%, and ΔTLG_3months_ 77%. There was no overall correlation between tumor-absorbed dose and change in FDG PET or ceCT parameters at the lesion level or significant difference in tumor-absorbed doses between metabolic responders and non-responders after treatment.

**Conclusion:**

Our analysis does not show any correlation between tumor-absorbed doses and changes in FDG PET or ceCT parameters for the included lesions. The combination regimen, including cold antibodies, may be one of the factors precluding such a correlation. Increased intra-patient response with increased tumor-absorbed doses was observed for most patients, implying individual variations in radiation sensitivity or biology.

**Trial registration:**

ClinicalTrials.gov Identifier (NCT01796171). Registered December 2012

**Supplementary Information:**

The online version contains supplementary material available at 10.1007/s00259-020-05098-x.

## Introduction

Targeted therapy with radiolabeled antibodies offers the unique possibility of dosimetric studies to plan treatment and to determine absorbed dose after treatment which other pharmacological treatment modalities lacks. While non-Hodgkin lymphomas (NHL) are known to be highly radiosensitive [1], dose-effect relationships known from external beam radiation cannot be directly applied to targeted radiotherapy [2, 3]. Developing methods to accurately determine tumor-absorbed doses and establishing dose-effect correlations are therefore important for radioimmunotherapy (RIT) development. However, attempts in previous studies to show reliable correlations between dosimetric evaluations and response after RIT in lymphoma have so far not been successful [4–7]. In the era of personalized precision medicine with development of highly advanced techniques, various metabolic and anatomical features have been made possible to measure. Thus new methods for dosimetry and imaging should be explored in this setting.

Two RITs have been approved by the US Food and Drug Administration: ^131^I-tositumomab (Bexxar^®^) and ^90^Y-ibritumomab tiuxetan (Zevalin^®^). Both agents consist of a monoclonal antibody specifically targeting the cell surface CD20 antigen, with a β-emitting radionuclide attached [8, 9]. Considering that these patients may be refractory to anti-CD20 monoclonal antibodies because of previous treatments, a conjugate that targets another B cell antigen, CD37 could be beneficial [10, 11]. Similar to CD20, CD37 is an antigen consistently expressed on the surface of B cells and B cell leukemia and lymphoma cells [10, 12, 13]. It is also found intracellularly at endosome and exosome level [14]. The B cell selectivity makes it a potentially valuable therapeutic target [10, 12, 13], and several CD37 reactive compounds have shown promise for treatment of NHL [12, 15–17]. Interestingly, preclinical studies have shown that the combined targeting of CD37 and CD20 with ^177^Lu-lilotomab satetraxetan and rituximab can improve the therapeutic outcome of NHL [18].

^177^Lu-lilotomab satetraxetan or Betalutin^®^ (Nordic Nanovector ASA, Oslo, Norway), which binds CD37, is a novel antibody-radionuclide conjugate for treatment of relapsed CD37+ indolent non-Hodgkin lymphoma which has been investigated in the first-in-human phase 1/2a study LYMRIT-37-01 [11]. Several regimens of pre-treatment with rituximab and pre-dosing with lilotomab (i.e., non-radioactive CD37 antibody) or rituximab and different activity levels of ^177^Lu-lilotomab satetraxetan were explored. We have already developed dosimetric methods to determine tumor-absorbed doses based on single photon emission tomography/computed tomography (SPECT/CT) data in this study [19]. 18F-fluorodeoxyglucose positron emission tomography/computed tomography (^18^F-FDG PET/CT) (from here on referred to as FDG PET) and contrast enhanced computed tomography (ceCT) were performed to evaluate response to treatment with 177Lu-lilotomab satetraxetan.

The aim of the present study was to investigate lesion-based response in the phase 1 part of the phase 1/2a LYMRIT 37-01 trial, exploring the changes in different metabolic and anatomical parameters in correlation with tumor-absorbed dose. FDG PET was used to define metabolic changes by the measures of maximum standardized uptake value (SUV_max_), metabolic tumor volume (MTV), total lesion glycolysis (TLG), and response according to a 5 point scale (Deauville criteria) in respective lesions at baseline and after 3 and 6 months. In addition, sum of perpendiculars measured by ceCT at baseline, 6 and 12 months was evaluated.

## Material and methods

### Patient characteristics and treatment

A total of 16 patients with relapsed B cell indolent NHL treated at Oslo University Hospital were included in this study. All were part of the multicenter LYMRIT 37-01 phase 1/2a study, but only patients from this center, with lesions eligible for dosimetry, were included to assure image standardization. CD37 status of patients was histologically confirmed. Histological subtypes were follicular lymphoma grades I and II and mantle cell lymphoma. All but one had received several previous treatment regimens including rituximab (Table 1). The phase 1/2a LYMRIT-37-01 trial was approved by the regional ethical committee, and all patients had signed an informed consent form.

**Table 1:** Patient characteristics

Characteristic	Value
Age (y), median (range)	70 (38–88)
Gender, *n* (%)	
Male/female	11 (69%)/5 (31%)
Histology, *n* (%)	
Follicular lymphoma, grade I	5 (31%)
Follicular lymphoma, grade II	10 (63%)
Mantle cell lymphoma	1 (6%)
Total injected activity, MBq	
Median (range)	1229 (746–2189)
Injected activity/body weight, *n* (%)	
10 MBq/kg	3 (19%)
15 MBq/kg	6 (38%)
20 MBq/kg	7 (44%)
Pre-treatment, *n* (%)	
Rituximab 375 mg/m^2^ 28 and 21 days before treatment (arms 1 and 2)	8 (50%)
Rituximab 375 mg/m^2^ 14 days before treatment (arms 3, 4, and 5)	8 (50%)
Pre-dosing, *n* (%)	
Lilotomab 40 mg (arm 1)	5 (31%)
No pre-dosing (arm 2)	3 (19%)
Rituximab 375 mg/m^2^ (arm 3)	2 (13%)
Lilotomab 100 mg/m^2^ (arm 4)	5 (31%)
Lilotomab 60 mg/m^2^ (arm 5)	1 (6%)
Number of tumors per patient, mod (range)	3 (1–5)
Number of previous treatments with rituximab, median (mod) (range)	10 (8) (0–26)

**Fig. 1:**
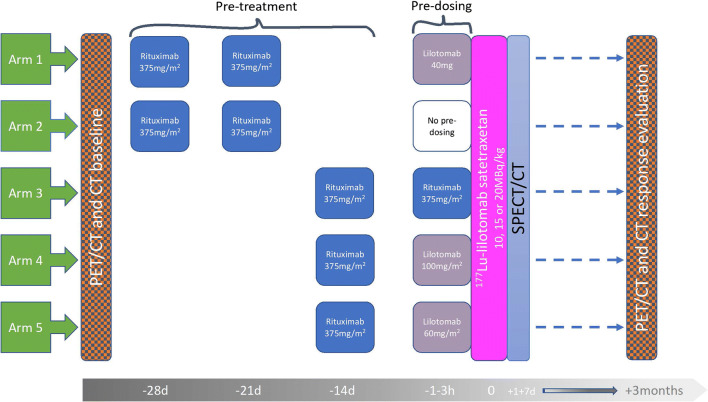
Study design: 3 different dosage levels, 10, 15, or 20 MBq/kg, were investigated in five arms of the phase 1/2a trial. Different pre-dosing regimens were given 1–3 h before ^177^Lu-lilotomab satetraxetan injection, except for arm 2. Pre-treatment regimens were given 28 and 21 days or 14 days before. FDG PET and ceCT were performed as baseline investigations and for response evaluation. The 0-h time point on the grey time line indicates administration of ^177^Lu-lilotomab satetraxetan

Different combinations of pre-treatment and pre-dosing regimens and three different dosage levels were tested in five arms. Patients received a single injection of ^177^Lu-lilotomab satetraxetan, either 10, 15, or 20 MBq/kg body weight. Before administration of ^177^Lu-lilotomab satetraxetan, all patients were pre-treated with rituximab (Fig. 1). In addition, patients in arm 1, 3, 4, and 5 received unlabeled antibody (lilotomab or rituximab) as pre-dosing 1–4 h before injection of ^177^Lu-lilotomab satetraxetan (Fig. 1).

### FDG PET and ceCT imaging

**Fig. 2:**
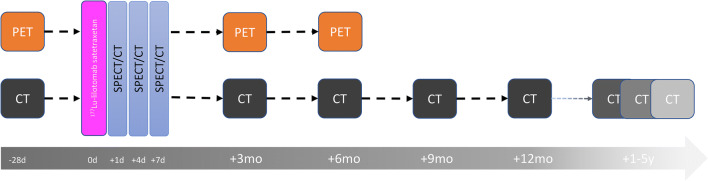
Imaging protocols: FDG PET was performed at baseline (PET_baseline_), within 2 weeks of the first pre-treatment. It was repeated for response evaluation at 3 months and at 6 months. Baseline ceCT was performed within 2 weeks of the start of pre-treatment and repeated at 3, 6, 9, and 12 months, 2–3 times after 1–2 years and once 2–5 years after ^177^Lu lilotomab satetraxetan treatment. SPECT/CT imaging was performed at days 1, 4, and 7 (expect for in arm 1, where only day 4 and 7 SPECT/CT was performed) and used for dosimetry calculations. The 0-h time point indicates administration of ^177^Lu-lilotomab satetraxetan

**Fig. 3:**
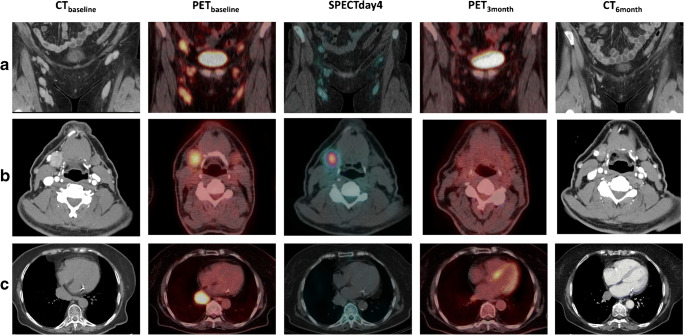
Images obtained at baseline and response evaluation, as well as SPECT/CT images showing the uptake of ^177^Lu-lilotomab satetraxetan at one time point. CT_baseline_, PET_baseline_, SPECT day 4, PET_3months_, and CT_6months_ for **a** patient 17, **b** patient 21, and **c** patient 16

FDG PET was performed at baseline (PET_baseline_); within 2 weeks of the first pre-treatment. It was repeated 3 months (PET_3months_) and 6 months (PET_6months_) after ^177^Lu-lilotomab satetraxetan treatment (Fig. 2). PET/CT images were acquired using a Siemens Biograph 16 or a GE Discovery MI PET/CT scanner. Acquisition was performed from vertex to mid-thigh 57–81 min after intravenous administration of 267 to 412 MBq of FDG, 3–2.5 min/bed scan time. All PET scans were reconstructed to comply with the EARL standard. Baseline ceCT was performed within 2 weeks of the first pre-treatment and repeated at regular time points after ^177^Lu-lilotomab satetraxetan treatment (Fig. 2). Only ceCT examinations at baseline (CT_baseline_), 6 months (CT_6months_) and 12 months (CT_12months_), were evaluated in the current work. Examples of lesions visualized on PET and ceCT are shown in Fig. 3. One patient did not undergo PET_6months_, CT_6months_, and CT_12months_ because of disease progression and change of treatment. Same applies to three other patients regarding CT_12month_.

### PET quantification

For each lesion eligible for tumor dosimetry, and with uptake higher than maximum standardized uptake value (SUV_max_) of liver at PET_baseline_, SUV_max_ and metabolic tumor volume (MTV) were measured, and total lesion glycolysis (TLG) was calculated at all PET time points, according to EANM procedure guidelines for tumor imaging: version 2 [20]. Syngo.via software solution (Siemens Healthineers) was used. A MTV threshold of 41% was applied. MTV and TLG at PET_3months_ and PET_6months_ were measured if uptakes were higher than liver uptake defined by PERCIST criteria [21]. Otherwise, they were registered as zero. SUV_max_ was registered as zero at PET_3months_ and PET_6months_ if the value was under blood background defined as in Deauville criteria [22]. Changes in FDG PET parameters in each lesion from PET_baseline_ to PET_3months_ were calculated as percent reduction from baseline value: ΔSUV_max-3months_, ΔMTV_3months_, and ΔTLG_3months_. Only SUV_max_ was used to measure metabolic change from PET_baseline_ to PET_6months_ (ΔSUV_max-6months_). All PET measurements and evaluations were performed by an experienced nuclear medicine physician.

### Response assessment

Response at lesion level was assessed at PET_3months_ according to Deauville criteria (5 point scale) [22]. Lesions were divided into 2 groups at PET_3months_: Deauville score 1, 2, and 3 defined as responders, PET_3months_ (−), and Deauville score 4 and 5 defined as non-responders, PET_3months_ (+). Uptake in lesions at PET_6months_ was similarly interpreted as negative, PET_6months_ (−), or positive, PET_6months_ (+).

Size of lesions on ceCT was assessed according to Lugano criteria by the sum of perpendiculars (SPD) [23]. Change in size from baseline to 6 months (ΔCT_6month_) and from baseline to 12 months (ΔCT_12months_) was calculated as percent reduction from baseline value.

Taking into account that rituximab given as pre-treatment may affect tumor volume before ^177^Lu lilotomab satetraxetan injection, a possible change in volume per lesion was defined. This was done by manual volume segmentation on the low-dose CT of the PET_baseline_ examination and the low-dose CT at day 1 SPECT/CT in arms 2, 3, 4, and 5. This enabled us to isolate the possible rituximab effect (up until day 1) from the investigational ^177^Lu-lilotomab satetraxetan effect. Change in volume (ΔCT_ritux_) was calculated as percent reduction from baseline value.

### SPECT/CT imaging and dosimetry

SPECT/CT scans were acquired with a Siemens Symbia T16 scanner at 96 and 168 h post-injection (p.i.) in arm 1 and at 24, 96, and 168 h p.i. in arms 2, 3, 4, and 5 (Fig. 2). The criteria for dosimetry to be performed included the ability to visually differentiate volume on low-dose CT and activity on SPECT, as well as a minimum volume of 1.5 mL. Two individual volume of interests (VOIs) were defined for each tumor, mass VOI delineated on low-dose CT and activity VOI delineated on SPECT, both drawn manually slice by slice by an experienced nuclear medicine specialist [19]. The masses derived from the CT VOIs were used for dose calculations, and the time activity curves based on uptake values at all time points was used to determine tumor-absorbed doses as described previously [19].

### Statistics

Pearson correlation tests were performed to investigate relationship between tumor-absorbed dose and ΔSUV_max-3months_, ΔMTV_3months_, ΔTLG_3months_, ΔSUV_max-6months_, ΔCT_6months_, and ΔCT_12months_. Pearson correlation coefficient with a significance level of *p* < 0.05 was used. The box plots show median values, interquartile ranges, and points lower or higher than 1.5 times the lower or upper quartile displayed as outliers. The Mann–Whitney *U* test was used to test differences in tumor-absorbed dose between response categories at PET_3months_ and PET_6months_. A null-hypothesis of equal populations with a rejection level of 0.05 was set. IBM SPSS statistics version 26 was used for all statistical analysis. GraphPad Prism 7 was used to create graphs.

## Results

Sixteen patients had one or more tumors eligible for dosimetry and had undergone PET_baseline_, PET_3months_ and PET_6months_. A total of 45 lesions were included for tumor dosimetry (1 to 5 lesions/patient). Tumor-absorbed doses ranged from 35 to 859 cGy (Table 2). Mean tumor-absorbed dose normalized for injected ^177^Lu-lilotomab satetraxetan was 2.3 mGy/MBq (range 0.4–6.7 mGy/MBq).

**Table 2:** Mean baseline PET parameters, tumor volume, tumor-absorbed dose, and tumor-absorbed dose/injected activity

• Arm	• Number of lesions	• Baseline PET parameters			• Tumor volume (mL)	• Tumor-absorbed dose [cGy]	• Tumor-absorbed dose/injected activity [mGy/MBq]
		• SUV_max_	• MTV	• TLG			
1	15	9.3 (5.3–13.9)	10.4 (2.1–21.3)	63.8 (9.2–132.3)	10,2 (1,5–25)	303 (76–794)	2.3 (0.5–5.3)
2	8	6,4 (4.6–12.1)	9.9 (3.9–15.6)	42.9 (12.2–92.9)	10.0 (3.8–15.7)	281 (123–728)	2.3 (0.9–5.1)
3	6	9.1 (6.3–12.3)	8.7 (1.9–26.6)	61.0 (7.6–220.8)	7.7 (1.7–20.4)	183 (35–287)	1.7 (0.4–2.7)
4	11	12.2 (7.2–19.4)	21.0 (2.6–112.8)	180.0 (16.7–1071.0)	28.4 (1.8–99.3)	370 (149–859)	2.6 (1.0–6.7)
5	5	11,6 (10.8–12.1)	7.7 (4.9–9.7)	54.8 (34.0–76.3)	7.1 (3.5–10.2)	482 (421–544)	2.8 (2.4–3.7)

Mean (range) values are given for each parameter

The mean baseline PET parameters for all lesions were SUV_max_ 9.7 (4.6–19.4), MTV 12.3 (1.9–112.8), and TLG 87.9 (7.6–1071.1). Mean tumor volume based on CT at day 4 SPECT was 14 mL (range 1.5–99.3 ml). An overview of the individual arms is given in Table 2. The mean reduction in PET parameters from baseline to 3 months were ΔSUV_max-3months_ 61% (−60 to 100%), ΔMTV_3months_ 80% (− 12 to 100%), and ΔTLG_3months_ 77% (− 70 to 100%) (refer to Supplementary Table 1 for individual lesions). The mean reduction in ΔSUV_max-6months_ was 56% (− 62 to 100%). The mean ΔCT_6months_ was 35% (− 41 to 72%), and ΔCT_12months_ was 33% (− 41 to 78%).

**Fig. 4:**
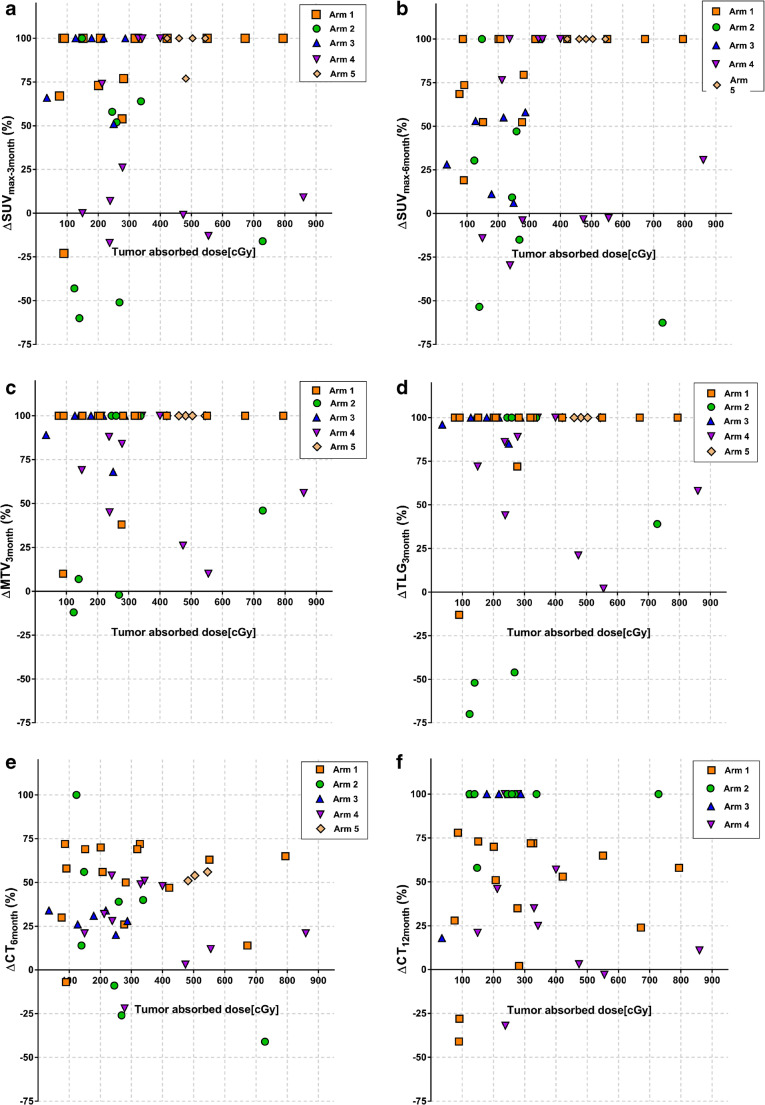
Tumor-absorbed doses plotted against **a** ΔSUV_max-3months_ (%), **b** ΔSUV_max-6months_ (%)_,_ **c** ΔMTV_3months_ (%)_,_ **d** ΔTLG_3months_ (%), **e** ΔCT_6months_ (%)_,_ and **f** ΔCT_12months_ (%). Each symbol represents a lesion, different symbols represents arms, as shown in legend. No clear overall correlations were found between tumor-absorbed doses and change in FDG PET and ceCT parameters

There was no overall correlation neither between tumor-absorbed doses and ΔSUV_max-3months_ (*r* = 0.54, *p* = 0.73), ΔSUV_max-6months_ (*r* = 0.11, *p* = 0.46), ΔMTV_3months_ (*r* = 0.02, *p* = 0.89), and ΔTLG_3months_ (*r* = 0.06, *p* = 0.66) nor between tumor-absorbed doses and ΔCT_6months_ (*r* = − 0.10, *p* = 0.53) and ΔCT_12months_ (*r* = 0.53, *p* = 0.8) (Fig. 4). The data set was also divided into 3 groups: Low lilotomab (arm 1), no lilotomab (arms 2 and 3), and high lilotomab (arms 4 and 5). Each group was analyzed separately, and no correlations between tumor-absorbed doses and ΔSUV_max-3months_ (low lilotomab *r* = 0.37, *p* = 0.17, no lilotomab *r* = − 0.21, *p* = 0.47, high lilotomab *r* = 0.07, *p* = 0.81), ΔSUV_max-6months_ (low lilotomab *r* = 0.45, *p* = 0.06, no lilotomab *r* = − 0.37, *p* = 0.19, high lilotomab *r* = 0.17, *p* = 0.54), ΔMTV_3months_ (low lilotomab *r* = 0.25, *p* = 0.38, no lilotomab *r* = − 0.06, *p* = 0.85, high lilotomab *r* = − 0.22, *p* = 0.42), and ΔTLG_3months:_ (low lilotomab *r* = 0.27, *p* = 0.33, no lilotomab *r* = − 0.01, *p* = 0.98, high lilotomab *r* = − 0.22, *p* = 0.41) were found.

Thirty of 45 tumors (67%) had a metabolic response at PET_3months_ according to the Deauville 5-point scale (Deauville 1, 2, and 3). Mean SUV_max_ at PET_baseline_ for responders was 9.2. Mean tumor volume was 9.8 mL. The mean reductions in FDG PET parameters for this group were ∆SUV_max-3months_ 91% (52%–100%), ΔMTV_3months_ 100%, and ΔTLG_3months_ 100%. Follow up with CT in this group showed mean reduction in SPD to be ΔCT_6months_ 47% (− 9 to 72%) and ΔCT_12months_ 47% (− 28 to 78%) (Supplementary Table 1).

Fifteen of 45 tumors (33%) were metabolic non-responders at PET_3months_ based on the 5-point scale (Deauville 4 and 5). Mean SUV_max_ at PET_baseline_ was 10.4. Mean tumor volume was 16.8 mL. Mean reductions in FDG PET parameters for this group were ∆SUV_max-3months_ – 0.6% (− 60 to 66%), ΔMTV_3months_ 41% (− 12 to 89%), and ΔTLG_3months_ 32% (− 70 to 96%). Mean reductions in SPD were ΔCT_6months_ 10% (− 41 to 54%) and mean ΔCT_12months_ 1.5% (− 41 to 35%) (Supplementary Table 1).

**Fig. 5:**
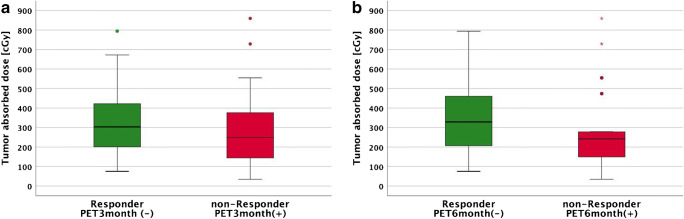
Tumor-absorbed doses for responders and non-responders: **a** Measured at PET_3months_. **b** Measured at PET_6months_

There was no statistically significant difference in tumor-absorbed doses between responding and non-responding lesions at PET_3months_ (*p* = 0.22) (Fig. 5a). Mean tumor-absorbed dose for responding lesions was 323 cGy (76–794 cGy), for non-responding lesions 313 cGy (35–859 cGy).

Metabolic response at PET_6months_ was also evaluated according to 5-point scale. Twenty-six of 44 tumors (59%) had metabolic response, and 18 of 44 tumors (41%) were metabolic non-responders at PET_6months_. Although there was slightly higher tumor-absorbed doses in responders than in non-responders at PET_6months_, this was not statistically significant (*p* = 0.18) (Fig. 5b). Mean absorbed dose for responding lesions was 342 cGy (76–794 cGy), while it was 291 cGy (35–859 cGy) for non-responding lesions.

Mean volume shrinkage per lesion in the interval from PET_baseline_ to ^177^Lu-lilotomab satetraxetan injection, defined as ΔCT_ritux_, was 8% (− 28 to 40%) (Supplementary Table 1). There was no statistically significant difference in ΔCT_ritux_ between arm 2 which received rituximab at 28 and 21 days before ^177^Lu-lilotomab satetraxetan and arms 3, 4, and 5 which received rituximab 14 days before (*p* = 0.55) (Fig. 1). Arm 3 was pooled with arms 4 and 5 as it is assumed that rituximab given as pre-dosing does not have any effect on tumor shrinkage as measured at CT the following day (day 1 SPECT/CT). No significant overall correlation was found between ΔCT_ritux_ and ΔSUV_max-3months_ (*r* = 0.32 *p* = 0.09) at PET_3months_. At PET_6months_, there was a significant correlation between ΔCT_ritux_ and ΔSUV_max-6months_ (*r* = 0.45 *p* = 0.02).

**Fig. 6:**
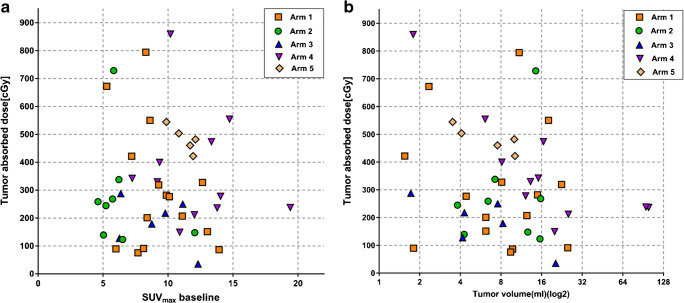
Baseline SUV_max_ **a** and tumor volume at start of treatment **b** plotted against tumor-absorbed dose. Tumor volume in **b** is plotted on a logarithmic scale. There was no correlation between baseline SUV_max_ or tumor volume and tumor-absorbed dose

Neither baseline SUV_max_ nor tumor volume (measured at the day 4 CT from the SPECT/CT scans) did show a correlation with tumor-absorbed dose (*r* = − 0.35, *p* = 0.82 and *r* = − 0.15, *p* = 0.31, respectively) (Fig. 6). Responding and non-responding lesions at PET_3months_ did not show any statistically significant differences in tumor volume or SUV_max_ at baseline (*p* = 0.21, *p* = 0.24, respectively) or at PET_6months_ (*p* = 0.96, *p* = 0.49, respectively).

## Discussion

Changes in FDG PET parameters after therapy with radiolabeled antibodies in correlation with tumor-absorbed doses have not been examined previously in patients with lymphoma. In this study, we tested different imaging parameters to evaluate response at the lesion level after ^177^Lu-lilotomab satetraxetan treatment and correlations with tumor-absorbed doses. No clear correlations were observed between the absorbed doses and the response based on imaging modalities and methods used.

**Fig. 7:**
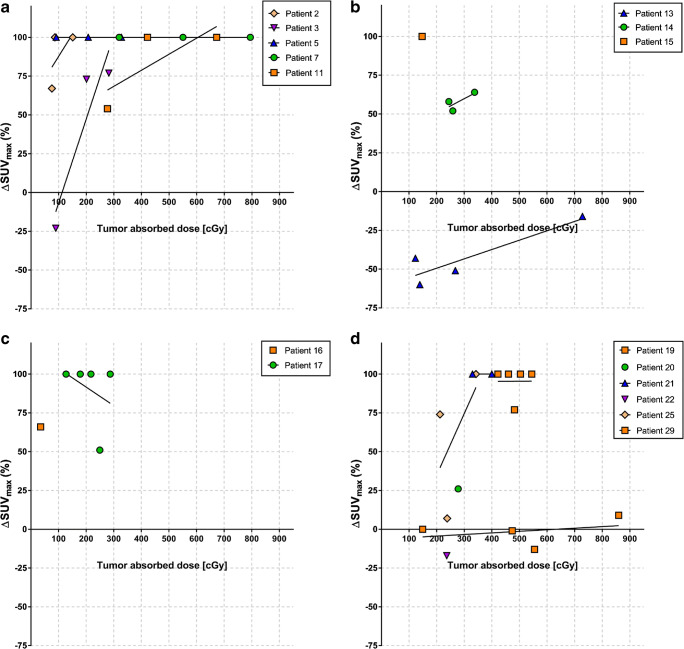
Tumor-absorbed doses plotted against ΔSUV_max-3months_ in **a** arm 1, **b** arm 2, **c** arm 3, and **d** arms 4 + 5. In each panel, different color and symbol codings represent individual patients, and each symbol represents an individual tumor lesion. The lines represent correlation between ΔSUV_max-3months_ and dose for tumors in individual patients. Interpatient variability in radiosensitivity is here evident, and intra-patient dose-response relationships also appear for most patients

NHL are known to be highly radiosensitive, and external beam radiotherapy (EBRT) is known to be very effective, and often curative in patients with localized disease [24]. Even low-dose radiation down to 4 Gy achieves high rates of local control [25]. However, current knowledge regarding absorbed dose-response relationships from EBRT cannot necessarily be directly applied to targeted radiotherapies [2]. Contrary to EBRT, targeted radiotherapies deliver continuous, generally low-dose rates, and often heterogeneous-absorbed doses to tissue. In the current study, large variations in tumor-absorbed doses were observed both intra-patient and within each study arm (Table 2), and the latter cannot be attributed only to different dosage levels (10–20 MBq/kg body weight). Tumor-absorbed doses in single lesions ranged from 35 to 859 cGy (median 330 cGy), and while the majority of lesions showed a metabolic response, no apparent overall dose-effect trend was observed (Fig. 4). The largest intra-patient variation found in our material was for patient 19, range 149–859 cGy (median 710 cGy) (Supplementary Table 1). Despite that the second highest mean absorbed dose was found in this patient, none of the four lesions responded at PET_3months_ and PET_6months_. The two other patients with the highest mean absorbed doses (patients 17 and 25) showed complete metabolic response at PET_3months_ and PET_6months_. Hence, there must be other factors that play important roles as discussed below, associated with the individual radiosensitivity in the current absorbed dose range. While no direct comparisons can be made, individual radiosensitivity is indicated by the occasionally observed responses for absorbed doses even less than the low-dose RT shown to be effective in NHL [25]. Furthermore, for the majority of cases, an improved response is observed for lesions receiving higher absorbed doses within individual patients (Fig. 7). Various gene products, as TP53, ATM, and NK-κB complexes, are known to be associated with radioresistance for lymphoma cells [26–30], and underlying gene mutations and expressions can potentially contribute to the interpatient variance in dose threshold needed for response. Correlating dosimetric results to efficacy measured by imaging modalities other than PET/CT has mostly been unsuccessful in previous studies of RIT in lymphoma [4–6]. Even though a larger data set of 124 lesions showed a significant correlation with response after ^131^I-tositumomab treatment; the *r* value between tumor shrinkage and mean tumor-absorbed dose was modest [7]. The authors measured both activity and tumor volume on SPECT/CT data, probably resulting in measurements comparable with our results [7]. We found absorbed doses for ^177^Lu-lilotomab satetraxetan therapy in the same order of magnitude as the absorbed doses in the mentioned study by Dewaraja et al. However, they reported a much stronger dose-response correlation for ^131^I-tositumomab treatment when adjusting for other biological effects, such as cold antibodies. These corrections were made possible by the tracer prediction studies performed for ^131^I-tositumomab patients and are therefore not possible to replicate in the current study.

FDG PET in evaluating metabolic response in lymphomas is widely accepted as the preferred imaging tool [31–33]. Still, use of this modality in response evaluation has met some skepticism for follicular lymphoma (FL)—contrary to Hodgkin lymphoma and diffuse large B cell lymphoma in the early PET era. Variations in glucose avidity of FL, response evaluation criteria with different thresholds, and use of older technology PET scanners may be have been contributing factors. However, a more recent study showed FDG PET as the most important factor predicting progression-free survival and overall survival after induction therapy for FL [34]. After the publication of the Lugano criteria, PET has become the standard imaging tool for staging, restaging, and response evaluation of lymphoma including FL [33]. We applied the widely accepted Deauville 5-point scale to categorize responders and non-responders [35, 36]. In our study, tumor-absorbed dose did not show significant overall correlation with change in FDG PET parameters. Interestingly, the difference in absorbed dose between responding and non-responding lesions became more apparent at PET_6months_ than at PET_3months_ (Fig. 5). Tumor-absorbed doses were slightly higher in responders than in non-responders at PET_6months_, although the difference was not significant (*p* = 0.18). Inflammation is a side effect of radiation on tissues, and it is also a well-known pitfall that inflammatory changes in irradiated tissue give higher FDG uptakes. It is possible that effects of RIT may be masked at PET_3months_ because of inflammation and become apparent after 6 months when the post irradiation inflammatory reaction is presumably over [37]. However, no overall correlation between tumor-absorbed doses and reduction in ΔSUV_max-6months_ was found. Fan et al. suggested SUV_max-liver_ based response evaluation to exclude a possible inflammatory-enhancing effect on FDG uptake. The group suggested a threshold of 1.6 × SUV_max-liver_ for interim PET and 1.4 × SUV_max-liver_ for end of treatment PET for better accuracy [35, 36]. In our cohort, this analysis failed to show any significant differences in tumor-absorbed doses between responders and non-responders at 3 months and 6 months (*p* = 0.9 and *p* = 0.64, respectively). Several studies found high false positive mid-therapy, response evaluation, and follow-up FDG PET studies in DLBCL patients receiving combination treatment with rituximab [38, 39]. This can be attributed to immune response activation, and according to Avivi et al., this effect may persist up to 3 years [38]. A meta-analysis by Sun et al. reported limited diagnostic accuracy of interim FDG PET with low-pooled sensitivity and specificity in NHL patients receiving R-CHOP (0.52 and 0.68, respectively) [40]. It is not unlikely that this inflammatory effect caused by immune response activation may mask treatment-related reduction in FDG uptake. To be consistent with previous investigations where volume derived from CT was used to assess response [5, 6], we also tested anatomical changes in ceCT from baseline to 6 and 12 months after therapy. No significant correlations with tumor-absorbed doses were found with this method neither.

Therapies with radionuclides linked to antibodies may also introduce immunological effects [3] that can be difficult to separate from radiobiological effects. Other factors than absorbed doses may therefore have influenced metabolic changes at FDG PET, like the pre-dosing and pre-treatment regimens tested (Fig. 1). From cell studies, the cell-killing effect of cold lilotomab appears to be limited [41], but the clinical translation is still to be determined. We have previously shown that pre-dosing with cold lilotomab results in lower absorbed doses to bone marrow and higher tumor-to-bone marrow absorbed dose ratios [42]. This demonstrates that the lilotomab pre-dosing alters biodistribution and probably renders more ^177^Lu-lilotomab satetraxetan available for tumors. While higher ΔSUV_max_ values were observed in the pre-dosed group (arms 1, 4, and 5) than in the not pre-dosed group (arms 2 and 3) (PET_3months_ *p* = 0.12, PET_6months_ *p* = .006), variances in tumor exposure and activity dosage levels can explain the differences. No correlations between tumor-absorbed dose and ΔSUV_max-3months_, ΔMTV_3months_, or ΔTLG_3months_ were found for groups separated by the amount of pre-dosing. However, with the limited numbers in each group, we cannot exclude the possibility that absorbed dose-response correlations are masked by different pre-dosing in this study.

Effect of rituximab given as pre-treatment may be another factor influencing change in FDG PET parameters. To investigate this possible effect, we have calculated the change in tumor size from manually determined volumes at the CT of the baseline PET/CT to the CT of day 1 SPECT/CT after ^177^Lu-lilotomab satetraxetan injection. Relatively large variations were observed (− 28 to 40%), independent of whether patients had received one rituximab injection 14 days before or two injections 21 and 28 days before. Previous studies have showed that treatment failure with rituximab increases with the number of previous rituximab treatments [43]; however, no significant correlation was found between number of previous rituximab treatments and ΔCT_ritux_ in our study (*r* = − 0.14, *p* = 0.47). These findings support that only limited volume shrinkage is likely to occur in the narrow time span of 2–4 weeks or that the results can be random since most of the included patients were heavily treated previously. Although a statistically significant correlation between the modest volume shrinkage caused by rituximab and ΔSUV_max-6months_ was observed, it is not plausible to assume that one or two CD20 antibody administrations would have had a long-lasting effect, especially since the 3 months data did not show any correlation.

In our study, neither baseline SUV_max_ nor initial tumor volume was correlated with tumor-absorbed dose (Fig. 6). Furthermore, there were no differences in baseline tumor volume or baseline SUV_max_ between responders and non-responders at PET_3month_ or PET_6month_. These results are of important clinical interest, as they demonstrate that prediction of tumor-absorbed doses or response is not feasible based on the imaging methods currently used in this study or timing of these. Development of a PET tracer based on the lilotomab antibody, made humanized to avoid HAMA, could provide more promising opportunities for imaging-based prediction.

## Conclusions

Overall dose-response relationship at the lesion level was not clearly demonstrated in this study of ^177^Lu-lilotomab satetraxetan treatment. FDG PET was used to evaluate the treatment-induced metabolic changes in our study, but ceCT changes were also investigated. The lack of correlation is in agreement with most dose-response studies of other radiolabeled antibodies. Baseline individual tumor volume and SUV_max_ probably do not have any predictive value for tumor-absorbed doses or metabolic response at the lesion level. Factors like variability in pre-treatment, pre-dosing, activity level, CD37 expression, as well as timing of FDG PET may also have played a role. Interestingly, we observed an intra-patient increased reduction in ΔSUV_max_ with higher absorbed doses. We hypothesize that variations in inherent radiosensitivity between patients and even tumors within patients may be as important as tumor-absorbed dose and baseline imaging characteristics to predict outcome after RIT. Hence, further investigations of genetic alterations correlated with radiosensitivity and resistance is warranted in order to better understand which patients will benefit most from treatment with ^177^Lu-lilotomab satetraxetan and other RIT compounds.

## Supplementary information

ESM 1 (DOCX 42 kb)
