# Supplementary material for: FDG PET/CT parameters and correlations with tumor-absorbed doses in a phase 1 trial of 177Lu-lilotomab satetraxetan for treatment of relapsed non-Hodgkin lymphoma
Source: Eur J Nucl Med Mol Imaging. 2020 Nov 16;48(6):1902–14. doi: 10.1007/s00259-020-05098-x (PMC8113302; doi:10.1007/s00259-020-05098-x)
Supplement: Supplementary file 1 — (DOCX 42 kb) [file 259_2020_5098_MOESM1_ESM.docx]

**Supplementary table 1** Patients, arms and individual lesion characteristics. Percent reduction in lesion volume before ^177^Lu-lilotomab satetraxetan treatment, ΔCT_ritux_, was attributed to rituximab effect. Percent reductions for FDG PET and ceCT parameters in individual lesions are shown.

| Arm | Patient | Lesion | Tumor absorbed dose (cGy) | Tumor volume  (mL) | ΔCT_ritux_  (%) | SUV_max-baseline_ | ΔSUV_max-3month_  (%) | ΔMTV_3month_  (%) | ΔTLG_3month_  (%) | ΔSUV_max-6month_  (%) | ΔCT_6month_  (%) | ΔCT_12month_  (%) | PET_3month_  ^a^(-/+) | PET_6month_  ^a^(-/+) |
| --- | --- | --- | --- | --- | --- | --- | --- | --- | --- | --- | --- | --- | --- | --- |
| 1 | 2 | 2a | 151 | 6,2 | NA | 13 | 100 | 100 | 100 | 42 | 69 | 51 | (-) | (+) |
| 1 | 2 | 2b | 87 | 9,8 | NA | 14 | 100 | 100 | 100 | 100 | 72 | -28 | (-) | (-) |
| 1 | 2 | 2c | 75 | 9,4 | NA | 8 | 67 | 100 | 100 | 69 | 30 | 72 | (-) | (-) |
| 1 | 3 | 3a | 201 | 6,2 | NA | 8 | 73 | 100 | 100 | 100 | 70 | 58 | (-) | (-) |
| 1 | 3 | 3b | 282 | 14,9 | NA | 10 | 77 | 100 | 100 | 80 | 50 | 65 | (-) | (-) |
| 1 | 3 | 3c | 90 | 1,8 | NA | 6 | -23 | 10 | -13 | 19 | -7 | 72 | (+) | (+) |
| 1 | 5 | 5a | 207 | 12,5 | NA | 11 | 100 | 100 | 100 | 100 | 56 | 24 | (-) | (-) |
| 1 | 5 | 5b | 90 | 25 | NA | 8 | 100 | 100 | 100 | 74 | 58 | 35 | (-) | (-) |
| 1 | 5 | 5c | 327 | 8,1 | NA | 13 | 100 | 100 | 100 | 100 | 72 | 53 | (-) | (-) |
| 1 | 7 | 7a | 794 | 10,9 | NA | 8 | 100 | 100 | 100 | 100 | 65 | 58 | (-) | (-) |
| 1 | 7 | 7b | 550 | 18,2 | NA | 9 | 100 | 100 | 100 | 100 | 63 | 65 | (-) | (-) |
| 1 | 7 | 7c | 320 | 22,5 | NA | 9 | 100 | 100 | 100 | 100 | 69 | 72 | (-) | (-) |
| 1 | 11 | 11a | 672 | 2,3 | NA | 5 | 100 | 100 | 100 | 100 | 14 | 24 | (-) | (-) |
| 1 | 11 | 11b | 277 | 4,4 | NA | 10 | 54 | 38 | 72 | 52 | 26 | 35 | (+) | (+) |
| 1 | 11 | 11c | 422 | 1,6 | NA | 7 | 100 | 100 | 100 | 100 | 47 | 53 | (-) | (-) |
| 2 | 13 | 13a | 268 | 15,7 | -11 | 6 | -51 | -2 | -46 | -15 | -26 | NA | (+) | (+) |
| 2 | 13 | 13b | 123 | 15,6 | 11 | 7 | -43 | -12 | -70 | 30 | NA | NA | (+) | (+) |
| 2 | 13 | 13c | 728 | 14,5 | -28 | 6 | -16 | 46 | 39 | -63 | -41 | NA | (+) | (+) |
| 2 | 13 | 13d | 139 | 4,3 | 11 | 5 | -60 | 7 | -52 | -54 | 14 | NA | (+) | (+) |
| 2 | 14 | 14a | 245 | 3,8 | -7 | 5 | 58 | 100 | 100 | 9 | -9 | NA | (-) | (+) |
| 2 | 14 | 14b | 259 | 6,4 | 27 | 5 | 52 | 100 | 100 | 47 | 39 | NA | (-) | (-) |
| 2 | 14 | 14c | 338 | 7,2 | 24 | 6 | 64 | 100 | 100 | 100 | 40 | NA | (-) | (-) |
| 2 | 15 | 15a | 147 | 12,6 | 28 | 12 | 100 | 100 | 100 | 100 | 56 | 58 | (-) | (-) |
| 3 | 16 | 16a | 35 | 20,4 | 16 | 12 | 66 | 100 | 100 | 28 | 34 | 18 | (+) | (+) |
| 3 | 17 | 17a | 179 | 8,3 | 7 | 9 | 100 | 100 | 100 | 11 | 31 | NA | (-) | (+) |
| 3 | 17 | 17b | 127 | 4,2 | 16 | 6 | 100 | 100 | 100 | 53 | 26 | NA | (-) | (-) |
| 3 | 17 | 17c | 250 | 7,6 | 8 | 11 | 51 | 68 | 85 | 7 | 20 | NA | (+) | (+) |
| 3 | 17 | 17d | 217 | 4,3 | -7 | 10 | 100 | 100 | 100 | 55 | 34 | NA | (-) | (+) |
| 3 | 17 | 17e | 287 | 1,7 | 2 | 6 | 100 | 100 | 100 | 58 | 28 | NA | (-) | (-) |
| 4 | 19 | 19a | 555 | 6,1 | -1 | 15 | -13 | 10 | 2 | -3 | 12 | -3 | (+) | (+) |
| 4 | 19 | 19b | 149 | 20 | 20 | 11 | 0 | 69 | 72 | -14 | 21 | 21 | (+) | (+) |
| 4 | 19 | 19c | 474 | 16,5 | 0 | 13 | -1 | 26 | 21 | -3 | 3 | 3 | (+) | (+) |
| 4 | 19 | 19d | 859 | 1,8 | 40 | 10 | 9 | 56 | 58 | 31 | 21 | 11 | (+) | (+) |
| 4 | 20 | 20a | 278 | 12,2 | 15 | 14 | 26 | 84 | 89 | -4 | -22 | ? | (+) | (+) |
| 4 | 21 | 21a | 400 | 8,1 | 3 | 9 | 100 | 100 | 100 | 100 | 48 | 57 | (-) | (-) |
| 4 | 21 | 21b | 330 | 13,2 | -6 | 9 | 100 | 100 | 100 | 100 | 49 | 35 | (-) | (-) |
| 4 | 22 | 22a | 236 | 99,3 | 5 | 14 | -17 | 88 | 86 | - | 54 | NA | (+) | NA |
| 4 | 25 | 25a | 238 | 94,9 | 4 | 19 | 7 | 45 | 44 | -30 | 28 | -32 | (+) | (+) |
| 4 | 25 | 25b | 343 | 15,2 | 1 | 7 | 100 | 100 | 100 | 100 | 51 | 25 | (-) | (-) |
| 4 | 25 | 25c | 212 | 25,3 | 26 | 12 | 74 | 100 | 100 | 77 | 32 | 46 | (-) | (-) |
| 5 | 29 | 29a | 422 | 10,2 | 12 | 12 | 100 | 100 | 100 | 100 | NA | NA | (-) | (-) |
| 5 | 29 | 29b | 482 | 10 | 16 | 12 | 77 | 100 | 100 | 100 | 51 | ? | (-) | (-) |
| 5 | 29 | 29c | 460 | 7,5 | 7 | 12 | 100 | 100 | 100 | 100 | NA | NA | (-) | (-) |
| 5 | 29 | 29d | 504 | 4,1 | 10 | 11 | 100 | 100 | 100 | 100 | 54 | ? | (-) | (-) |
| 5 | 29 | 29e | 544 | 3,5 | 12 | 10 | 100 | 100 | 100 | 100 | 56 | ? | (-) | (-) |

^a^Response at lesion level was assessed by PET according to 5 point scale (Deauville criteria,) and lesions were divided into 2 groups as PET (-) (Deauville score 1,2 and 3) and PET (+) (Deauville score 4 and 5).
